# Full-flexion gap balancing-driven personalized preoperative planning for TKA: biomechanical evidence of improved load symmetry in an in vitro model

**DOI:** 10.1186/s42836-026-00402-w

**Published:** 2026-06-05

**Authors:** Zijun Tang, Diyang Zou, Yangyang Yang, Nan Zheng, Zhemin Zhu, Juan Wang, Pingyue Li, Rongshan Cheng, Guoqiang Zhang, Tsung-Yuan Tsai

**Affiliations:** 1 School of Biomedical Engineering and Med-X Research Institute, Shanghai Jiao Tong University, Shanghai, 200030 China; 2 Engineering Research Center for Digital Medicine of the Ministry of Education, Shanghai, 200030 China; 3 Guangdong Key Lab of Orthopedic Technology and Implant, General Hospital of Southern Theater Command of PLA, Guangzhou, 510010 China; 4 General Hospital of People’s Liberation Army, Beijing, 100141 China; 5 Department of Joint Surgery, Hebei Medical University Third Hospital, Shijiazhuang, 050051 China

**Keywords:** Total knee arthroplasty, Gap-balanced, Dynamic dual X-ray, Preoperative optimization, In vitro experiment

## Abstract

**Purpose:**

Conventional morphology-based planning (MB) in total knee arthroplasty (TKA) often results in imbalanced medial–lateral loading, which is a major risk factor for prosthesis loosening. This study aimed to evaluate a full-flexion gap balance planning (GB) approach and compare its biomechanical performance with MB planning.

**Methods:**

TKA surgical planning was performed on eight subjects. In vivo knee kinematics were obtained using a dual X-ray imaging system. The implant position was optimized within an in silico surgical-planning framework by minimizing medial–lateral gap differences across the full flexion range using in vivo dynamic kinematics. Using 3D-printed in vitro models, joint contact forces were measured at various flexion angles to compare medial–lateral load distribution between GB and MB planning.

**Results:**

GB planning optimized femoral varus/valgus by approximately 4° within anatomical constraints, significantly reducing the medial–lateral gap difference to a mean of < 1 mm across the entire flexion range (*p* < 0.05). In contrast, the medial–lateral gap difference exceeded 2 mm with MB planning. Furthermore, at mid-to-high flexion angles (30°–100°), GB planning reduced the medial–lateral load imbalance by approximately 38%–67% compared with MB planning (*p* < 0.05).

**Conclusion:**

GB planning significantly improves medial–lateral load balance across the full range of flexion compared to conventional MB planning. This personalized planning optimizes joint loading patterns in vitro, which may theoretically align with principles aimed at reducing flexion-related adverse outcomes.

## Introduction

Total knee arthroplasty (TKA) is a primary treatment for end-stage knee osteoarthritis, and its clinical demand continues to grow. It is projected that by 2050, the incidence of TKA will increase by approximately 130% compared with current levels, with associated additional annual healthcare expenditures exceeding €1.1 billion [1, 2]. In conventional surgical practice, implant positioning largely depends on the surgeon’s experience, and malalignment or deviation of the mechanical axis are major contributors to postoperative knee instability, prosthesis loosening, and other complications [3, 4]. Preoperative planning for TKA has therefore become a key step in the current clinical workflow.

Mechanical alignment (MA) has historically served as the conventional reference strategy in TKA, aiming to achieve a neutrally aligned knee by referencing the hip–knee–ankle (HKA) mechanical axis [5, 6]. However, recent advances in robotic-assisted TKA and patient-specific alignment philosophies have broadened the available surgical options, and the optimal alignment strategy for clinical outcomes remains under debate [7, 8]. Conventional gap-balancing techniques are used to create rectangular, symmetric gaps at full extension and at 90° of flexion to maintain joint stability [9, 10]. However, studies suggest that achieving balance at 0° and 90° does not necessarily maintain balanced behavior throughout the full flexion range, and residual mid-flexion laxity or load shifts may still occur [11–13]. In addition, morphology-based measured resections may lead to unpredictable femoral component rotation and condylar lift-off [14, 15]. This mismatch between intraoperative static assessment and postoperative dynamic functional demands is considered a key contributor to residual pain and low functional satisfaction in some patients [16]. Although computer navigation and pressure-sensing technologies have improved the detection of such imbalance, a planning approach that can achieve gap balance across the full flexion range is still lacking in current clinical practice.

This study used in vivo flexion kinematics acquired using a dual X-ray imaging system as the input for in silico planning. An in vitro experiment was performed to compare postoperative biomechanics between full-flexion gap-balanced (GB) planning and conventional morphology-based (MB) planning in TKA. This workflow may help mitigate biomechanical risk factors associated with flexion instability and persistent pain.

## Methods

### In vivo data collection

**Fig. 1:**
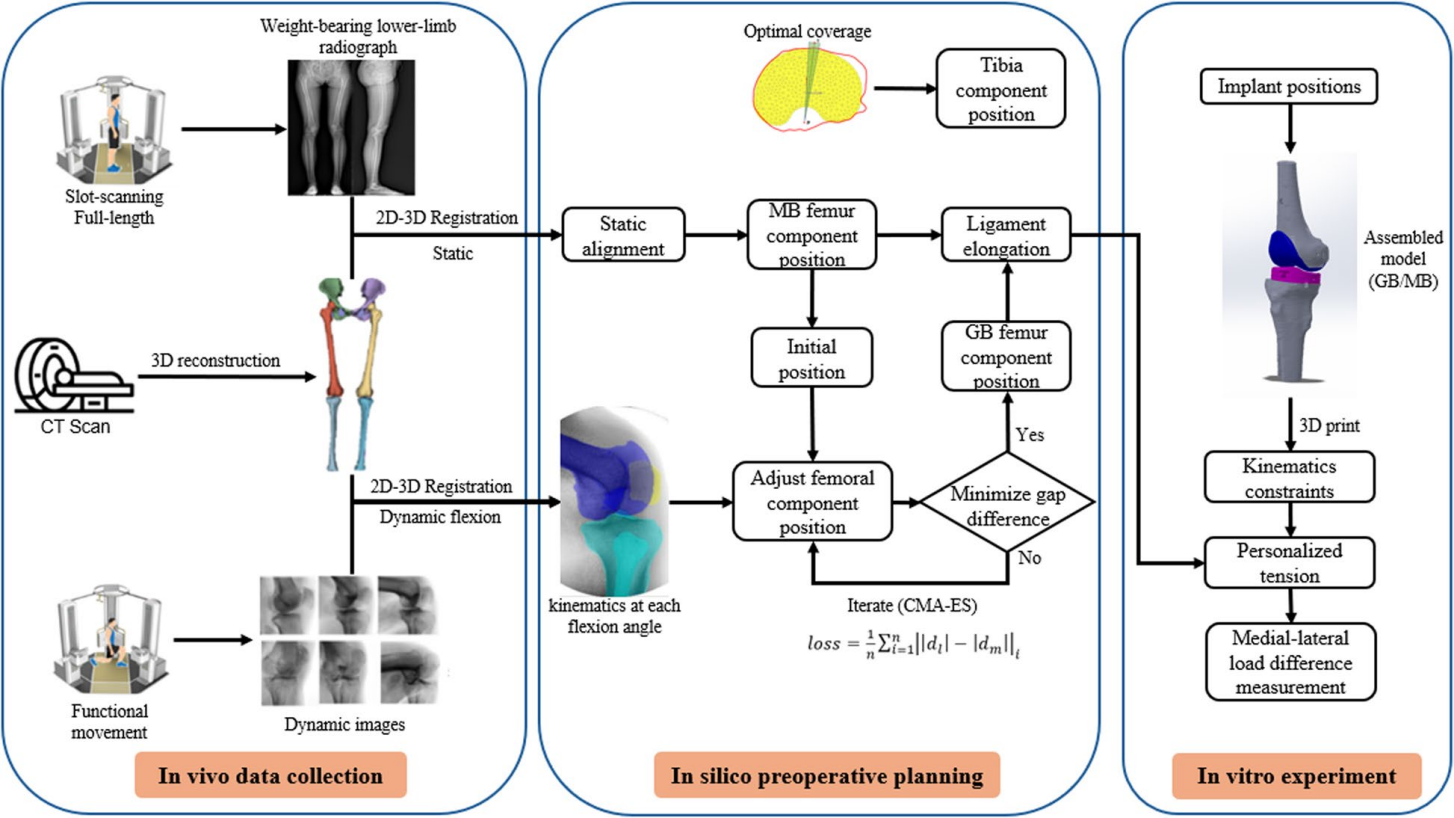
Overview of the study workflow. MB: morphology-based planning. GB: gap balance planning

Eight subjects (1 male, 7 females; age 70.9 ± 3.4 years; BMI 27.6 ± 1.9 kg/m2) performed a weight-bearing lunge (0°–100°) under a dual X-ray imaging system (Eagle Eye, TAOiMAGE, Shanghai, China). All subjects underwent unilateral total knee arthroplasty. Inclusion criteria were end-stage primary osteoarthritis, mild varus deformity, and passive flexion greater than 90°. Subjects with rheumatoid arthritis, post-traumatic arthritis, severe valgus deformity, or a history of knee arthroplasty were excluded. CT was used for 3D model reconstruction (SOMATOM Definition AS1; Siemens, Germany, 120 kV, and 80 mA). 3D kinematics were obtained using a custom 2D–3D registration pipeline (Fig. 1) [17, 18]. The study was approved by the institutional review board and ethics committee (Shanghai Sixth People’s Hospital, approval No. 2023-KY-071), and written informed consent was obtained from all participants.

### In silico preoperative planning

#### Tibial component position planning

Simulated tibial osteotomy was performed using a custom program. The same workflow was applied to both MB and GB plans. The tibial cut matched the native posterior slope, with a resection depth of 8 mm below the lateral plateau. A simulated annealing search (max iterations = 200, initial temperature = 5) [19] was used to match the implant undersurface contour to the resection contour and identify the component position that maximized tibial coverage. Tibial rotation was constrained within ± 5° of the Insall line, and the maximum allowable overhang was limited to 2 mm [20]. The position of the tibial component was adjusted to maximize the penalized tibial coverage index (PCI), which accounted for both tibial surface coverage and implant overhang (Fig. 1). The PCI was defined as:

$$\frac{{A}_{I}\cap {A}_{T}-Sub({A}_{I},{A}_{T})}{{A}_{T}}\times 100\%$$

Formula 1. A_I_ denotes the region enclosed by the underside of the implant; A_T_ denotes the tibial resection surface area. Sub (A_I_, A_T_) represents the remaining region after subtracting area A_T_ from area A_I_.

#### Femoral component position planning

Inputs for femoral component positioning were derived from weight-bearing full-length lower-limb radiographs and CT-based bone models. The HKA mechanical axis measured from the radiographs was used for varus-valgus correction. The three-dimensional standing pose obtained by registering the radiographs to the CT bone models was also used as the initial position for planning. On this basis, the MB plan was defined as a morphology-matched preoperative reference based on a measured-resection concept. Distal and posterior resections were set to match the corresponding component thickness. The posterior resection plane was aligned parallel to the transepicondylar axis [21, 22].

GB planning used subject-specific flexion kinematics as input constraints to determine the relative pose between the femoral component and the tibial resection plane at each flexion angle. The femoral component position obtained from MB planning was used as the initial estimate. A search was performed around this initial pose while ensuring anatomically feasible component placement. The shortest distances from the medial and lateral condylar surfaces of the femoral component to the tibial resection plane were defined as the postoperative medial and lateral gaps. The optimization objective was to minimize the mean medial–lateral gap difference across flexion angles by adjusting femoral component position. Changes in the optimized femoral component pose relative to the initial MB pose were quantified in Six Degrees of Freedom. The output included the optimized femoral component position and the corresponding distal femoral resection plane. Based on the computed joint gaps, the tibial insert thickness most suitable for each subject was selected as the individualized recommendation. The loss function was defined as:

$$loss=\frac{1}{n}{\sum\nolimits}_{i=1}^{n}{\left|\left|{d}_{l}\right|-\left|{d}_{m}\right|\right|}_{i}$$

Formula 2. n denotes the number of three-dimensional joint positions used for optimization during motion, ${\left|\left|{d}_{l}\right|-\left|{d}_{m}\right|\right|}_{i}$ representing the absolute medial–lateral gap difference at each flexion position.

The covariance matrix adaptation evolution strategy (CMA-ES) was used to solve for the optimal relative position of the femoral component [23]. This method converges reliably in high-dimensional, anatomically constrained search spaces and is less likely to get trapped in a local minimum. In addition, to evaluate the influence of the target flexion range, two reduced-range GB variants were included. In the standing-only strategy, the optimization minimized the medial–lateral gap difference only at 0° flexion. In the standing-plus-90° strategy, the optimization minimized the mean medial–lateral gap difference at 0° and 90° flexion. These two variants were included to assess the performance differences between balancing over a limited set of flexion angles and the full-flexion GB planning.

#### Ligament length and tension simulation

After virtual implantation of the components into the subject-specific bone models using the optimized position, a postoperative bone-implant geometric model was generated. For the native knee, ligament length at each flexion angle was calculated on the subject-specific native bone models before virtual implantation. Under the guidance of an experienced surgeon, the femoral and tibial attachment sites of the anterior and posterior bundles of the superficial medial collateral ligament (aMCL and pMCL) and the intermediate bundle of the lateral collateral ligament (iLCL) were identified on the 3D model. A ligament wrapping-path algorithm was then used to compute the path length of each bundle across the full flexion range. At each flexion angle, an optimization algorithm was used to determine the shortest 3D wrapping path of each ligament bundle around the bone and implant surfaces, and the path length was defined as the ligament bundle length [24]. Ligament length at 0° flexion was used as the reference length. Changes in ligament tension were derived from the flexion-dependent variation in ligament elongation $\varepsilon$, enabling a subject-specific mechanical loading protocol. Ligaments exhibit nonlinear mechanical behavior, and the relationship between ligament force and strain can be expressed as:

$$f=\left\{\begin{array}{c}0.25k\cdot \frac{{\varepsilon }^{2}}{{\varepsilon}_{1}}, 0\le \varepsilon \le 2{\varepsilon}_{1},\\ k\left(\varepsilon -{\varepsilon}_{1}\right), \varepsilon>2{\varepsilon}_{1},\\ 0, \varepsilon <0,\end{array}\right.$$

Formula 3. The ligament strain constant is denoted by ε₁, with a value of 0.03. The ligament stiffness is denoted by k. The k values for the aMCL, pMCL, and iLCL were 2500 N, 3000 N, and 2000 N, respectively [25, 26].

### In vitro experimental setup

**Fig. 2:**
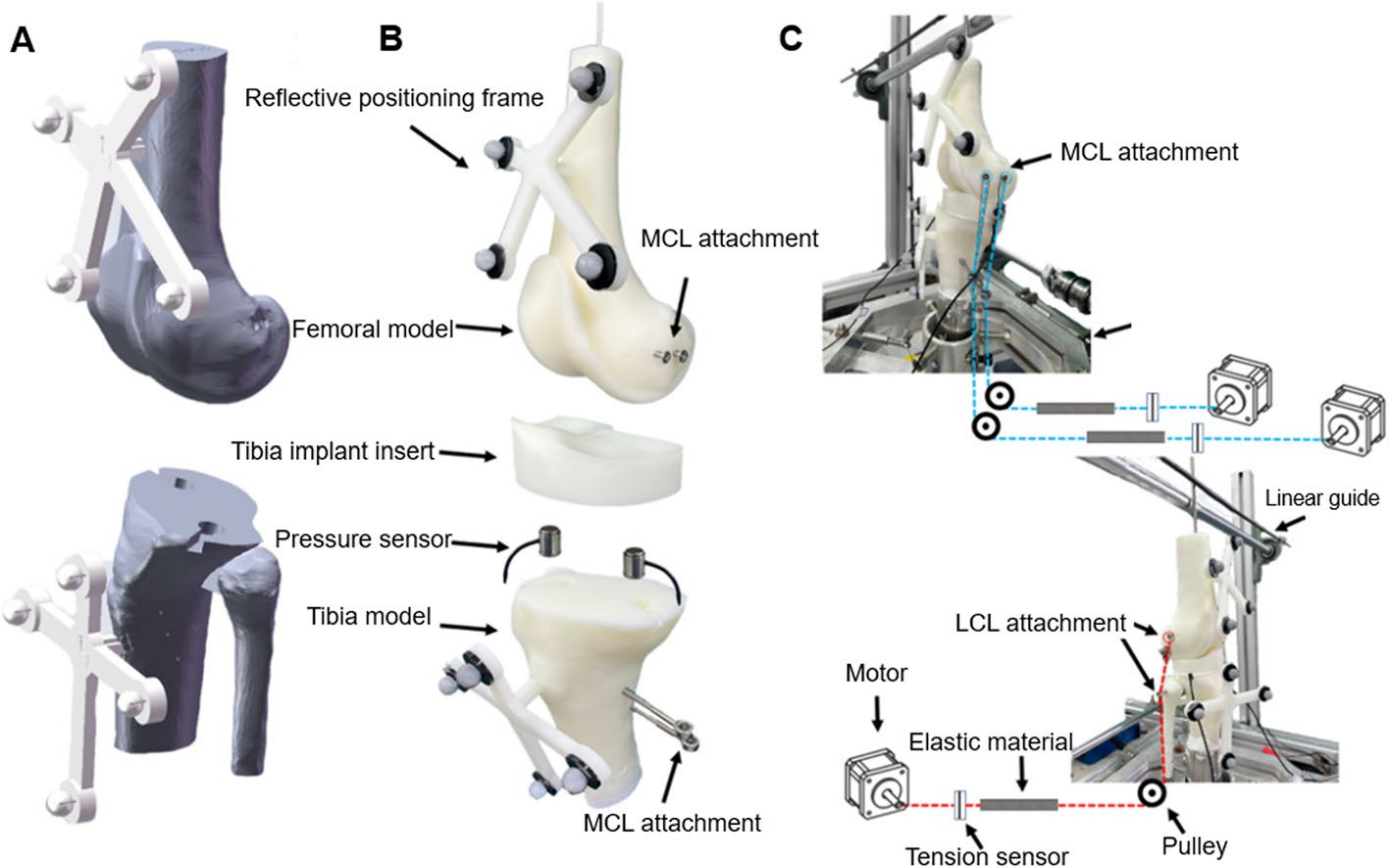
In vitro experimental setup. **A** Digital model of femoral component implantation and tibial osteotomy; **B** Assembly illustration of the TKA knee joint model; **C** Illustration of in vitro testing

In vitro experiments were performed using assembled 3D printed models (Lite 800, Union Tech) in photosensitive resin. Two pressure sensors were embedded in both the medial and lateral regions of the tibial model to measure compartmental contact pressures. Preoperative flexion kinematics were corrected for varus-valgus based on each subject’s standing alignment. The corrected kinematics were used as the input. A previously validated in vitro testing platform capable of applying loads with an error of less than 2 N was used in this study (Fig. 2) [27–29]. In this study, the tibial component was rigidly fixed to the base. Anterior–posterior and medial–lateral translations were constrained by articular surface contact [30]. Femoral flexion and internal–external rotation were constrained by a guide rail. The remaining degrees of freedom were allowed to self-equilibrate. The motors pulled the cables anchored to screws at the ligament attachment sites to apply the prescribed ligament tensions. Motor actuation was controlled by a LabVIEW-based closed-loop program to maintain the target tensions in real time (Fig. 2). Baseline tensions were set to 130 N medially and 100 N laterally [27]. On this basis, the precomputed tensions of the aMCL, pMCL, and iLCL were simultaneously applied to their corresponding bundles, and the final applied tension was defined as the sum of the baseline tension and the corresponding precomputed ligament tension. Measurements were performed at multiple predefined flexion angles to represent the full flexion range (0°–100°, sampled every 10°).

### Statistical analysis

The Shapiro–Wilk test was used to verify the normality of variables. Although normality was not violated, nonparametric tests were used given the small sample size. Friedman tests were used to compare ligament lengths among the two planning strategies and original length, followed by pairwise comparisons with the paired Wilcoxon signed-rank test and Holm–Bonferroni correction to control for multiple testing. Joint loads at each flexion angle were directly compared using the paired Wilcoxon signed-rank test, and Holm–Bonferroni correction was applied across all *p*-values for multiple angles. Statistical significance was set at *p* < 0.05. All analyses were conducted in MATLAB (MathWorks, USA). A sensitivity analysis was performed using G*Power (version 3.1.9.7). With *N* = 8, α = 0.05, and power = 0.80, the study had 80% power to detect a large effect size (Cohen’s $d\ge 1.15$), suggesting adequate sensitivity for large between-plan differences. Effect sizes were also calculated at each flexion angle to quantify the magnitude of the observed differences.

## Results

**Fig. 3:**
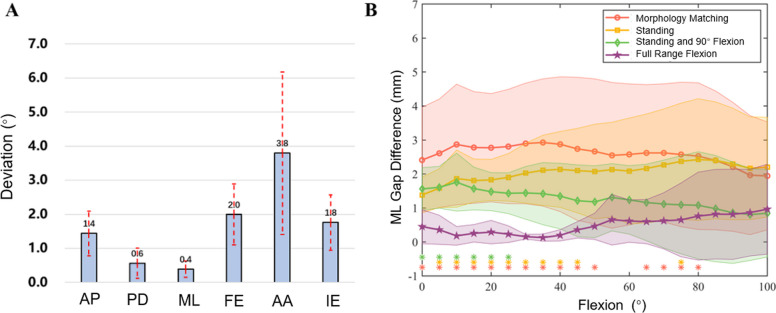
Implant positions and medial–lateral gap differences under different planning. **A** Difference in femoral component position; **B** The difference in the gap values between the medial–lateral sides of the knee joint. AP: anterior–posterior translation, PD: proximal–distal translation, ML: medial–lateral translation, FE: flexion–extension rotation, AA: abduction–adduction rotation, IE: internal–external rotation

The penalized tibial coverage index reached 77.1% ± 5% under anatomical constraints. The component’s AP axis deviated from the Insall line by 3.0° ± 1.1°. Compared to the MB plan, the GB plan primarily adjusted the femoral varus–valgus orientation by 3.8° ± 2.4° (range: 0.7°–7.0°) (Fig. 3A). Other directional deviations remained minor.

The GB plan achieved superior mediolateral symmetry throughout the entire flexion range compared to both the MB plan and the reduced-range GB variants. The MB plan resulted in gap differences exceeding 2 mm at all angles. The standing strategy still showed substantial residual asymmetry, with medial–lateral gap differences remaining around 2 mm or higher across most of the flexion range. The standing and 90° flexion strategy partially improved the balance, reducing the gap difference to approximately 1–2 mm. In contrast, the GB plan maintained a highly balanced profile with a mean difference below 1 mm (Fig. 3B). Statistically, the GB plan produced significantly smaller gap differences than the MB strategy from 0° to 80° (*p* < 0.05, with effect sizes ranging from 0.99 to 2.20). It also outperformed the standing-plus-90° strategy at low flexion angles between 0° and 25° (*p* < 0.05, with effect sizes ranging from 1.98 to 2.55).

**Fig. 4:**
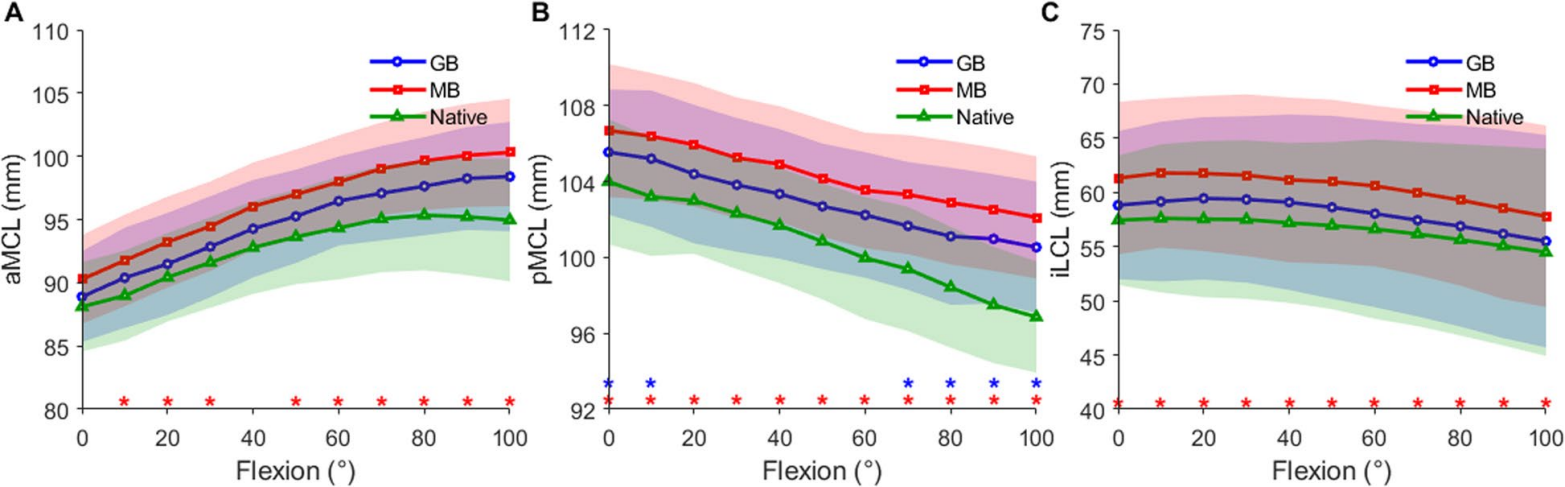
Ligament length changes from 0° to 100° of flexion in the native knee and after TKA under the two planning strategies. **A** aMCL length; **B** pMCL length; **C** iLCL length. The lines represent the mean values. The shadows indicate one standard deviation. Red asterisks indicate significant differences between ligament lengths under MB planning and the native reference, whereas blue asterisks indicate significant differences between ligament lengths under GB planning and the native reference (*p* < 0.05)

Overall, while both planning strategies captured the general kinematic trends of the native state, the GB plan demonstrated closer fidelity to native ligament lengths. Specifically, within the medial collateral ligament complex, both aMCL and pMCL lengths followed their respective native patterns (Fig. 4A&B). The native aMCL length increased from 88.1 ± 3.5 mm at 0° to 95.1 ± 4.2 mm at 70° before plateauing, whereas the native pMCL shortened from 104.0 ± 3.3 mm at 0° to 96.9 ± 2.9 mm at 100°. Despite these shared directional trends, the MB plan exhibited persistent and significant deviations from native lengths across the entire flexion range (*p* < 0.05, with effect sizes ranging from 0.79 to 0.89). In contrast, the GB plan maintained closer biomechanical alignment, with significant deviations limited only to specific or extreme flexion angles (aMCL: 10° and 80°–100°; pMCL: 0°–10° and 70°–100°).

The iLCL length in the native knee showed a slight decrease during flexion (57.4 ± 6.0 mm at 0° to 54.5 ± 9.6 mm at 100°). The GB plan closely mimicked this native pattern; however, the MB plan exhibited a distinct “stable-then-decrease” profile, remaining unchanged until 50° before declining, which resulted in a significant departure from native kinematics across the entire flexion arc (*p* < 0.05, with effect sizes ranging from 0.79 to 0.89; Fig. 4C).

**Fig. 5:**
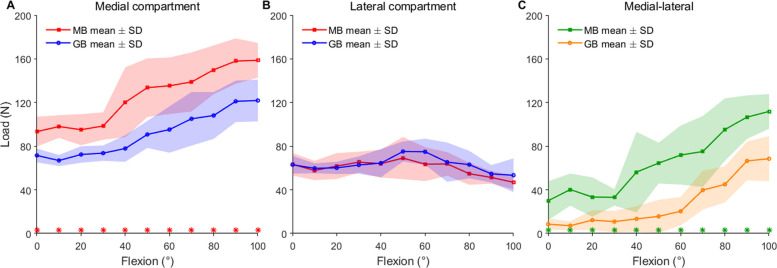
Medial–lateral load under the MB plan and GB plan. **A** Medial compartment load; **B** Lateral compartment load; **C** Load difference between medial and lateral compartments. The lines represent the mean values. The shadows indicate one standard deviation. Asterisks indicate significant differences between MB and GB planning at the corresponding flexion angles (*p* < 0.05)

Regarding medial compartment loads, both plans remained relatively stable during early flexion before significantly increasing toward high flexion. Specifically, the MB plan exhibited consistently higher medial loads across the entire flexion range compared to the GB plan (*p* < 0.05, with effect sizes ranging from 0.89 to 1.00). For the GB plan, loads were stable from 0° to 40° (0°: 72 N and 40°: 78 N) and reached 122 N at 100°. In contrast, the MB plan remained stable only until 30° (0°: 93 N and 30°: 99 N) before rising to 159 N at 100° (Fig. 5A). In terms of lateral compartment forces, no significant differences were observed between the two plans, with both maintaining similar magnitudes throughout the flexion range (Fig. 5B).

The medial–lateral load difference increased with flexion from mid-to-high flexion angles for both strategies (Fig. 5C). However, the GB plan maintained a more balanced profile, showing significantly smaller medial–lateral differences than the MB plan from 30° to 100° (*p* < 0.05, with effect sizes ranging from 0.89 to 1.00). For instance, at 100° of flexion, the medial–lateral difference was 69 N for the GB plan, compared to 112 N for the MB plan. Although the shaded standard deviation regions were relatively large at some flexion angles, this finding reflected substantial inter-subject variability in baseline anatomy and ligament-related loading responses, further supporting the need for personalized preoperative planning.

## Discussion

This study integrated in vivo kinematics, in silico simulation, and in vitro experiments. For the first time, in vivo dynamic kinematics were used to enable GB planning. Joint gaps and load distribution were compared between GB and MB planning across 0°–100° of flexion. Compared with the MB plan, the GB plan optimized femoral varus-valgus alignment by an average of approximately 4°. A varus outlier of this magnitude can lead to a 14-fold increase in the risk of revision [31]. Although the GB plan effectively improved full-flexion gap balance, the relatively large femoral varus–valgus adjustment observed in some cases should be interpreted with caution. Future implementation in robotic-assisted TKA software may require predefined coronal alignment safe zones to prevent excessive varus–valgus deviation and joint-line obliquity. The GB plan reduced the medial–lateral gap difference to < 1.0 mm across the entire flexion range, whereas the MB plan maintained a gap difference > 2.0 mm. This geometric improvement translated into load sharing. At mid-to-high flexion angles (30°–100°), the medial–lateral load difference under MB ranged from 33 to 112 N. Under the GB plan, it decreased to 11–69 N. In addition, the GB plan more closely reproduced native collateral ligament length patterns (aMCL: 88.9–98.4 mm; pMCL: 105.6–100.5 mm; iLCL: 58.8–55.5 mm), which may help restore knee proprioception [32]. By contrast, the MB plan showed a distinct iLCL pattern, with lengths remaining nearly constant before 50° of flexion. These results suggest that GB planning improves gap and load balance across the full flexion range, which may reduce the risk of related adverse outcomes [16].

Clinically, soft-tissue balance in TKA is often interpreted as achieving equal medial–lateral compartment loads across the flexion range [27]. Conventional gap balancing relies heavily on the surgeon’s subjective assessment, and even after standard balancing steps, up to 74% of knees still require further adjustment [33]. Previous studies have suggested that the medial–lateral intercompartmental load difference should be maintained within 15 lb (≈66.7 N) [34]. In our study, the medial–lateral intercompartmental load difference under the GB plan remained within this recommended range up to 80° of flexion, indicating a more stable medial–lateral load distribution. In contrast, under the MB plan, the medial–lateral load difference exceeded 60 N at flexion angles of around 40°, showing a larger load asymmetry at mid-flexion. Existing evidence suggests that patients with better load balance during flexion tend to experience greater pain relief and higher postoperative satisfaction [16]. Thus, the proposed planning method may help reduce intraoperative adjustments and improve surgical efficiency, although the clinical relevance of the observed load differences still requires confirmation in future clinical studies.

Inadequate pain relief and reduced patient satisfaction after TKA are often associated with excessive medial laxity in mid-flexion [35]. Compared with MB planning, the GB plan more closely reproduced the native collateral ligament recruitment pattern across 0°–100° of flexion, which may reduce the risk of soft-tissue over-tensioning in flexion [36, 37]. This more native-like tension distribution is expected to maintain medial stability in mid-flexion while improving postoperative functional recovery [38].

From a biomechanical perspective, the optimal pattern of medial–lateral balance throughout flexion remains controversial [35]. Some studies support replicating the “medially tight, laterally lax” characteristic of the native knee [39], whereas others argue that maintaining relatively balanced medial–lateral compartment loads after TKA may improve implant longevity [40, 41]. In this study, minimization of medial–lateral gap differences over the full flexion range was adopted as the planning objective, and the measured peak resultant joint load was approximately 250 N. This load level is comparable to the axial compressive forces commonly used in previous in vitro contact mechanics studies of the native and TKA knees and can therefore be used to compare the effects of different balancing strategies on knee joint mechanics [42]. Future work may incorporate alternative objective functions for further comparison.

This study has some limitations. First, the in vitro model used in this study did not fully incorporate active muscle forces, cruciate ligaments, or other complex soft-tissue constraints. The MB model used in this study represented an unadjusted morphology-based geometric baseline. In real TKA, surgeons routinely perform intraoperative soft-tissue releases to compensate for medial–lateral imbalance. This surgical process was not captured in the present rigid comparative model. Future studies may incorporate cadaveric experiments to evaluate the interactions among implant positioning, soft-tissue constraints, and joint mechanics under more physiologic conditions, thereby providing stronger evidence to support clinical translation of this planning strategy. Second, current robot-assisted systems have demonstrated bone-resection accuracy better than 1 mm [21, 22]. Therefore, the GB planning outputs proposed in this study do not exceed the precision limits of current technology. However, more work is needed to determine compatibility with existing robotic platforms and to clarify what modifications would be required for intraoperative implementation. Third, the in vitro models were 3D printed using photosensitive resin, whose friction coefficient and elastic modulus differ from those of native cartilage and clinical TKA implant materials. Accordingly, the measured compartmental loads primarily reflect relative differences between the two planning strategies and should not be regarded as absolute representations of clinical joint forces. Fourth, the ligament loading model used in this study estimated ligament tension based on a nonlinear spring model and did not account for the native viscoelastic properties of ligamentous soft tissues. In addition, the in vivo kinematics were derived from a weight-bearing lunge task, which may not fully represent the range of postoperative daily activities. Finally, the present analysis was limited to immediate biomechanical outcomes under controlled in vitro conditions. Although the observed differences in load distribution may reflect a more favorable short-term mechanical environment, the study did not address time-dependent processes such as implant wear, bone remodeling, or aseptic loosening, which depend on cumulative postoperative loading and long-term implant–bone interactions.

## Conclusion

Personalized GB planning reduced medial–lateral load imbalance by approximately 38%–67% at mid-to-high flexion angles compared with MB planning. It also improved gap symmetry and yielded collateral ligament length changes that were closer to the native reference derived from in vivo kinematics across 0°–100° of flexion. These improvements suggest that this strategy optimizes joint loading and soft-tissue conditions in vitro, which may theoretically support biomechanical strategies intended to reduce flexion-related adverse outcomes.

## Data Availability

The raw imaging data used in this study are not publicly available because they are derived from human participants and are subject to ethical and privacy restrictions. Processed data supporting the findings of this study are available from the corresponding author upon reasonable request.

## Declarations

### Code availability

The custom code used for image-based planning and biomechanical analysis is not publicly posted because it contains research-specific implementation details. Relevant code supporting the main findings of this study is available from the corresponding author upon reasonable request.
